# Hematological malignancies in East Africa—Which cancers to expect and how to provide services

**DOI:** 10.1371/journal.pone.0232848

**Published:** 2020-05-06

**Authors:** Steven Alan Leak, Lilian Gasper Mmbaga, Elifuraha Wilson Mkwizu, Priscus John Mapendo, Oliver Henke

**Affiliations:** Cancer Care Centre, Kilimanjaro Christian Medical Centre, Moshi, United Republic of Tanzania; University of Ghana College of Health Sciences, GHANA

## Abstract

**Background:**

Sub-Saharan Africa (SSA) has an increasing non-communicable disease burden. Tanzania has an incidence of more than 35,000 cancer cases per year with an 80% mortality rate. Hematological malignancies account for 10% of these cases. The numbers will double within the next 10 years due to demographic changes, better diagnostic capabilities and life style changes. Kilimanjaro Christian Medical Centre established a Cancer Care Centre (CCC) in December 2016 for a catchment area of 15 million people in Northern Tanzania. This article aims to display the hematological diagnosis and characteristics of the patients as well as to describe the advancements of hematologic services in a low resource setting.

**Methods:**

A cross-sectional analysis of all hematological malignancies at CCC from December 2016 to May 2019 was performed and a narrative report provides information about diagnostic means, treatment and the use of synergies.

**Results:**

A total of 209 cases have been documented, the most common malignancies were NHL and MM with 44% and 20%. 36% of NHL cases, 16% of MM cases and 63% of CML cases were seen in patients under the age of 45. When subcategorized, CLL/SLL cases had a median age was 56.5, 51 years for those with other entities of NHL. Sexes were almost equally balanced in all NHL groups while clear male predominance was found in HL and CML.

**Discussion:**

Malignancies occur at a younger age and higher stages than in Western countries. It can be assumed that infections play a key role herein. Closing the gap of hematologic services in SSA can be achieved by adapting and reshaping existing infrastructure and partnering with international organizations.

## Introduction

We live in an increasingly interconnected, global community with a fast-growing population. On one hand, we see rapid advances in healthcare as a result of global cooperation, while on the other hand, disparities in health care are becoming more apparent. Sub Saharan Africa has an exponentially increasing healthcare need; currently estimated to have 25% of the global disease burden. In addition to health stressors including HIV/AIDS and resurgent epidemics; Africa also faces an ageing population, and an increasing non-communicable disease burden [1,2].

In 2008 the incidence of cancer cases in Africa was estimated to be 681,000 with a mortality of 512,000 [3]. Without considering changes in incidence rates, projections suggest that these figures are likely to rise to 1,27 million and 970,000 respectively by 2030 [3]. In Tanzania alone, more than 35,000 new cancer cases per year are reported, with a mortality rate reaching nearly 80% [4]. Hematological malignancies including Hodgkin lymphoma (HL), Non-Hodgkin lymphoma (NHL), leukemia and Multiple Myeloma (MM) currently account for approximately 10% of these cases [5].

Kilimanjaro Christian Medical Centre (KCMC) based in Northern Tanzania with predominantly rural areas and two main urban centers, Moshi and Arusha. Until 2016, the majority of diagnosed malignancies were referred to the governmental Ocean Road Cancer Institute (ORCI), located in the 550 km distant city of Dar Es Salaam, for their ongoing management and care. As a result, loss to follow up and presentations at late stage were significant problems. Recognizing the needs, KCMC established its own Cancer Care Centre (CCC) in December 2016 to provide accessible service to the catchment population. The centre consists of two buildings containing a small laboratory, two consultation rooms, a procedure room, 16 outpatient chemotherapy bays, waiting area and two administrative offices.

KCMC harbors one of three cancer registries in Tanzania, the other two being based at ORCI, and Bugando Medical Centre in Mwanza. These databases used to rely mostly on diagnosis made by the respective Pathology Departments, hence hematological malignancies diagnosed by other means including polymerase chain reaction (PCR), karyotyping, flow cytometry and/or blood smear cytology are not well documented. As a result of these shortcomings and other factors, reliability of epidemiological cancer data, and of hematological cancer data in particular, can be considered as weak [6].

This paper should serve two purposes: First, to describe the various hematological malignancy cases which have presented to CCC and the associated clinical and demographic factors. Secondly, to highlight the challenges in managing these cases in a resource limited setting as well as providing solutions by displaying our approaches for the improvement of diagnostics, treatment and overall patient care.

## Methods

### Study setting

CCC is based in the city of Moshi within the Kilimanjaro region in Northern Tanzania. The catchment area of this Department consists of the regions Kilimanjaro, Tanga, Manyara, and Arusha with a total population of approximately 15 million. Despite the two urban centres Arusha City and Moshi, the area can be described as rural. CCC is accessible through the main road of the country, connecting the cities in Northern Tanzania with the economical center of Tanzania Dar Es Salaam in the East, Arusha and Mwanza in the West and the capital of Tanzania, Dodoma, in the South. The transport infrastructure outside the main routes are mainly gravel roads and impose difficulties to travel, especially during the rainy season.

### Study period and design

We conducted a cross-sectional analysis of all hematological malignancies from the cancer registry of CCC from its establishment in December 2016 until May 2019.

A convenient sampling of all recorded cases of hematological malignancies in CCC´s cancer registry has been applied. The collected data were: Diagnosis, age at time of diagnosis, sex and stage of the disease (where available). Diagnosis were categorized in to 6 main groups: HL, MM, NHL, chronic myeloid leukemia (CML), acute myeloid leukemia (AML), and acute lymphoblastic leukemia (ALL) including staging, sex and age at diagnosis. NHL cases were further subcategorized into the most common entities, diffuse large cell lymphoma (DLCL), Chronic lymphocytic leukemia/Small lymphocytic lymphoma (CLL/SLL) and other NHL cases. Median age at diagnosis and interquartile range (IQR) were calculated for each group, the sex ratio and clinical staging were also displayed.

A descriptive report provides information about the development of diagnostic means, and treatment advances through cooperation and using synergies of existing structures.

### Ethical considerations

Ethical clearance was granted by the Kilimanjaro Christian Medical College Research Ethics and Review Committee in accordance with the Declaration of Helsinki. Informed consent was waived because of the retrospective nature of the study and the analysis used anonymous clinical data.

## Results

During the report period, 209 cases of hematological malignancies were documented in the cancer registry of the CCC. Table 1 shows the cases by age and sex distribution. Table 2 shows the sub-categorization of NHL cases. The most common malignancies seen were NHL and MM accounting for 44% and 20% of cases respectively. 36% of NHL cases, 16% of MM cases and 63% of CML cases were seen in patients under the age of 45. When cases of NHL cases were subcategorized the median age was 56.5 for those with CLL/SLL and 51 years for those with other entities of NHL. Sexes were almost equally balanced in all NHL groups while clear male predominance was found in HL and CML.

**Table 1:** Hematological malignancies, subcategorized by entities and age groups.

Age	Hodgkin Lymphoma	Non-Hodgkin Lymphoma	Multiple Myeloma	AML	ALL	CML
Total (209)	18	92	43	15	17	24
0–14	2	3	0	2	7	2
15–24	7	9	3	2	9	0
25–44	6	21	4	7	0	13
45–64	3	36	25	4	1	9
65+	0	23	11	0	0	0
Median age at diagnosis	23.5	54	58	35	15	42
IQR	10 (18.3–28.3)	25.5 (38–63.5)	13.5 (51.5–65)	21.5 (21–42.5)	13 (4–17)	17.5 (35.5–53
male:female ration	2.6:1	1.1:1	1.3:1	1.1:1	1.8:1	2.4:1

**Table 2:** CLL/SLL, DL(B)CL and other NHL categorized by age groups.

Age	CLL/SLL	DL(B)CL	Other Non-Hodgkin Lymphoma
Total (92)	40	10	42
0–14	0	0	3
15–24	4	1	4
25–44	8	3	10
45–64	16	4	16
65+	12	2	9
Median age at diagnosis	56.5	54.5	51
IQR	28.5 (40–68.5)	19.8 (39.8–59.5)	30.8 (31.8–62.5)
male:female ratio	1:1	1.5:1	1.1:1

**Fig 1:**
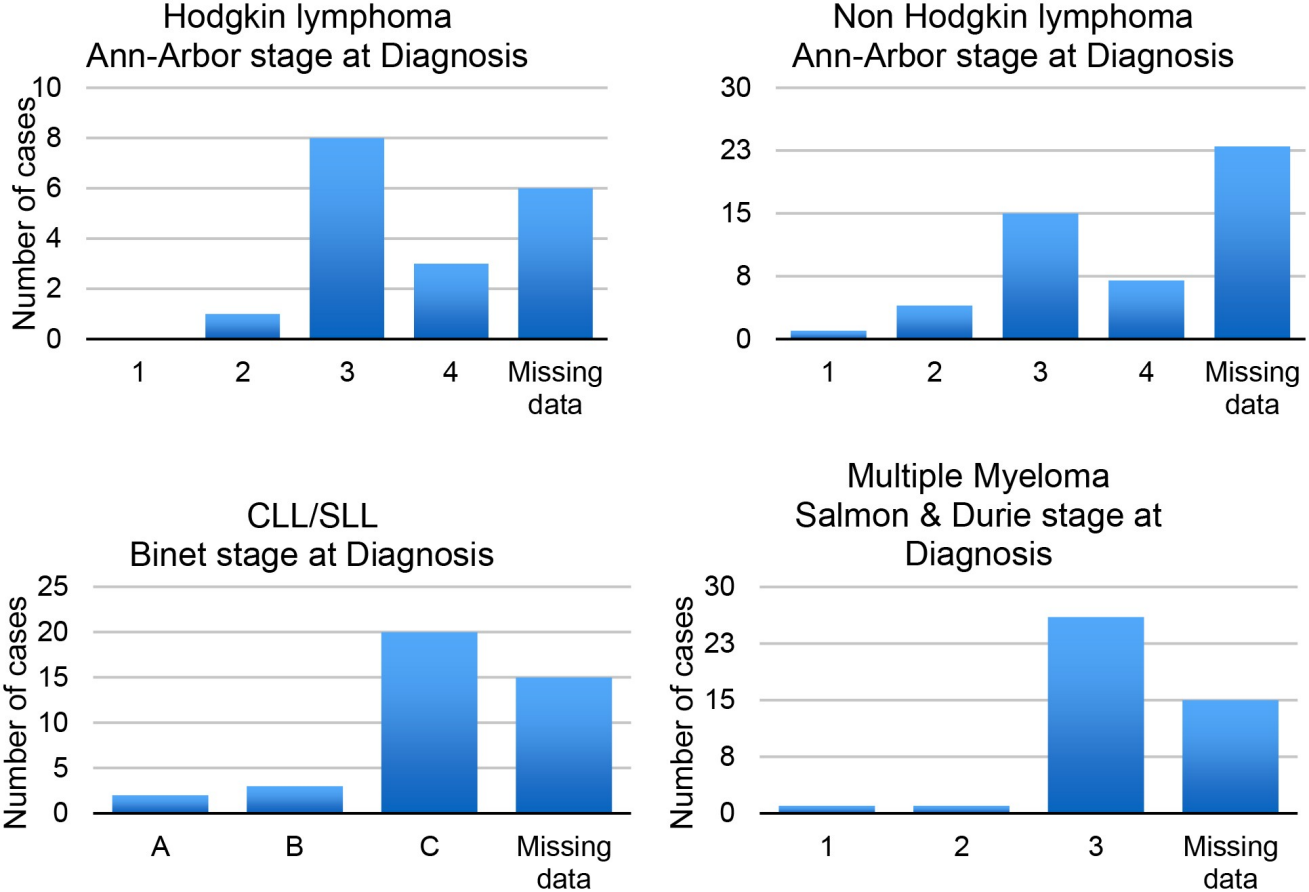
Ann-Arbor staging for Hodgkin and Non-Hodgkin lymphoma cases, Binet stage for CLL/SLL cases and Salmon & Durie stage for MM cases at diagnosis.

**Fig 2:**
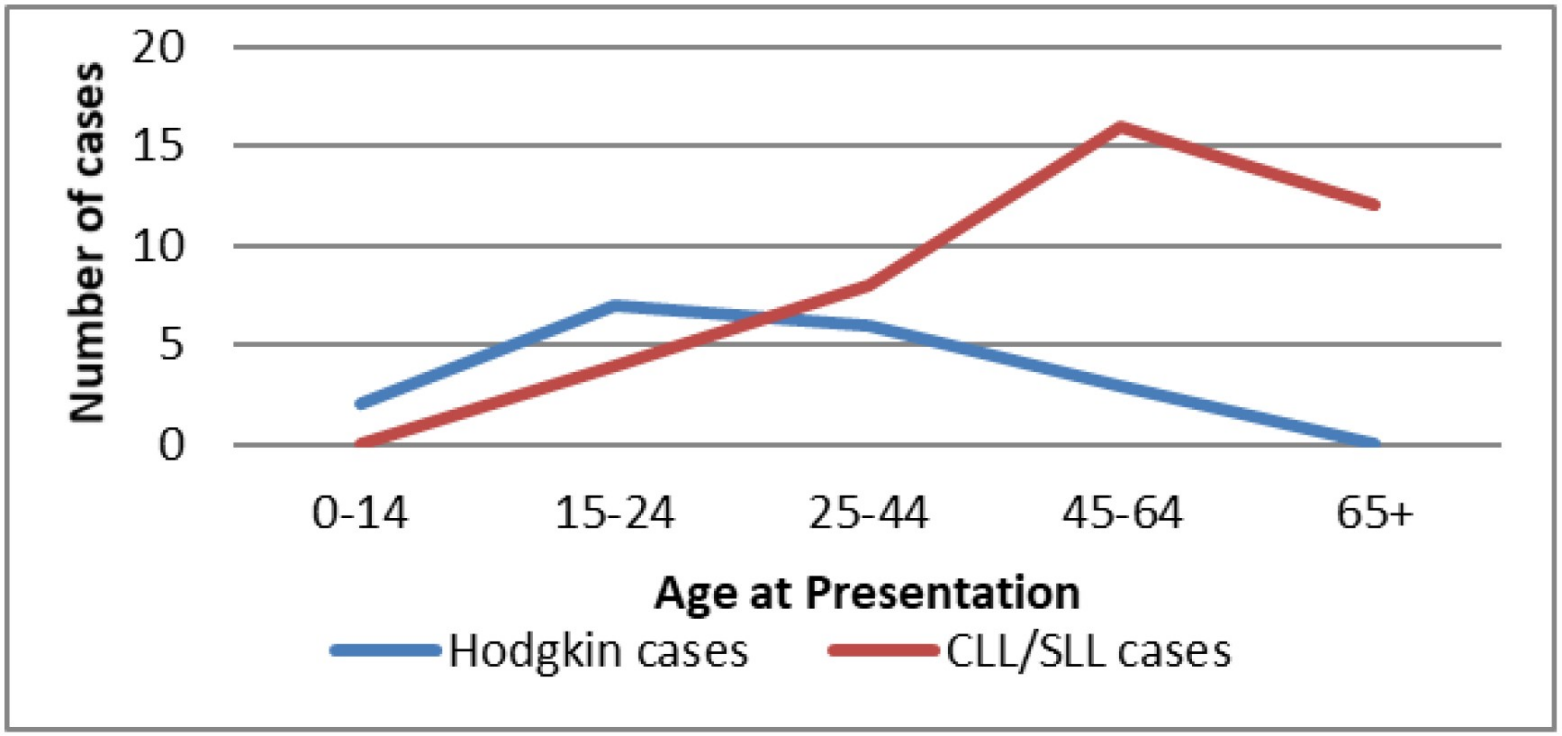
Cases of Hodgkin lymphoma and CLL/SLL per age category December 2016—May 2019.

The majority of cases were in stage 3 or 4 (according to Ann-Arbor classification) and in stage 3 according to Binet and Salmon and Durie classification at the time of diagnosis (Fig 1). Fig 2 demonstrates that age distribution of Hodgkin lymphoma and CLL/SLL cases.

### Establishing the Cancer Care Centre

Initiated by the Foundation for Cancer Care in Tanzania (FCCT), a US based non-governmental organization, funds were allocated to KCMC for the construction of two buildings; an outpatient clinic and an infusion centre, that represent the KCMC Oncology Department (CCC). Due to sparse specialized staff resources in the field of Hematology and Oncology, a KCMC nurse was sent to Duke University Oncology Department (North Carolina, USA), for specialized training in administering chemotherapy in early 2016. A Tanzanian Oncologist and Radiation Specialist, trained in South Africa, was recruited through FCCT to lead the Centre. Another specialized doctor (Hematology and Medical Oncology) and a Public Health officer were recruited as development aid workers from a German Lutheran Mission (Mission Eine Welt) and a Tanzanian Clinic Coordinator was employed. These workers represented the workforce before the Centre was officially opened, to plan the integration of the new Department into the structure of the hospital, write up therapy protocols and standard operation procedures, explore procurement pathways and public relation, and to discuss prevention campaigns strategies. Supported by various short-term volunteers from abroad, that focused on hands-on training for additional KCMC staff that was subsequently allocated to the new Centre.

**Fig 3:**
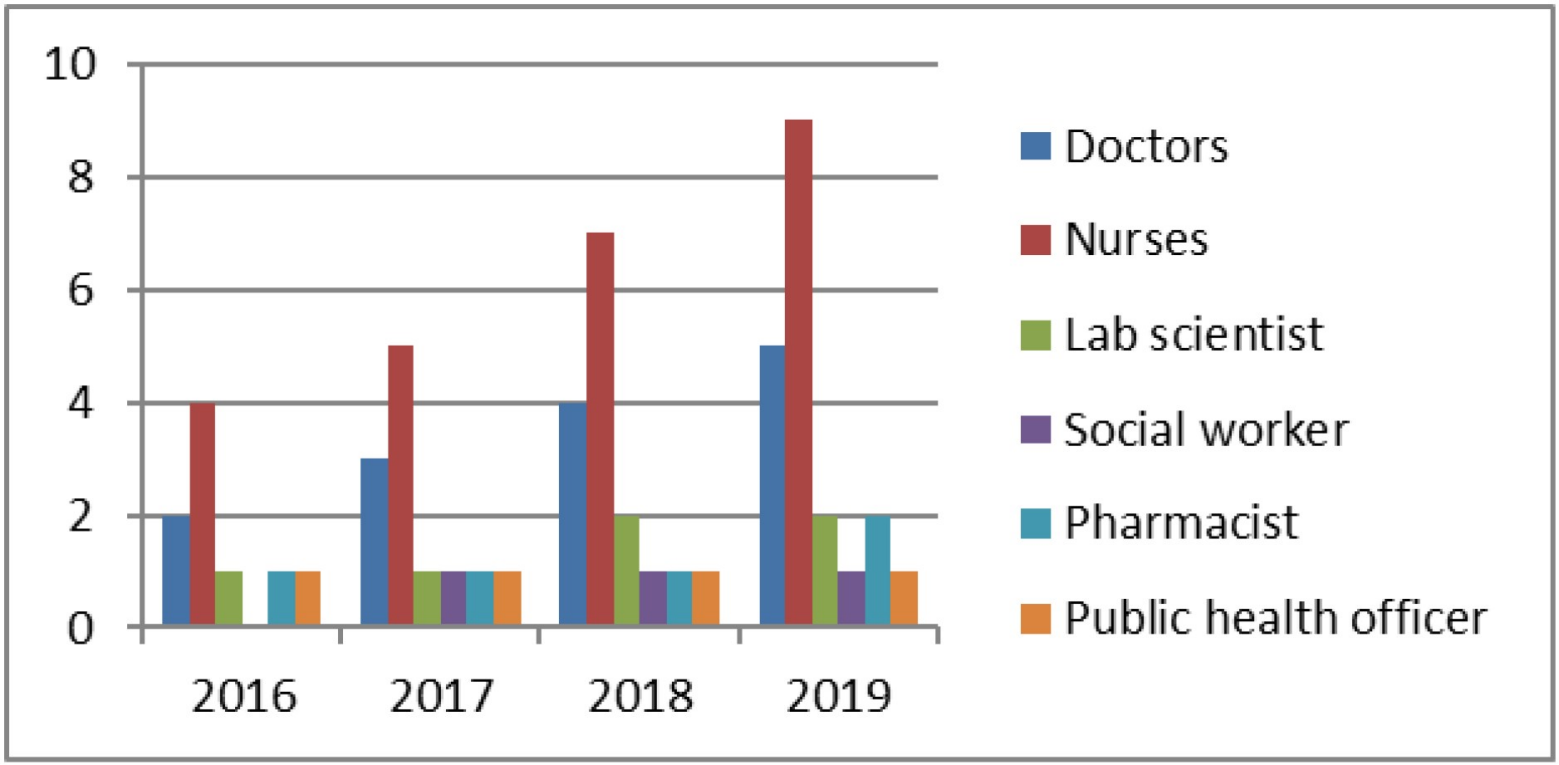
Development of permanent staff at the CCC since its establishment.

At the time of opening, 3 additional nurses and a pharmacist were permanently allocated. Fig 3 displays the further staff development in the following years with the addition of assistant doctors (registrars), a second specialist in Hematology, social worker and additional administrative staff.

As part of the tertiary hospital KCMC, radiology and pathology diagnostics and a main hospital laboratory were available from the beginning, even though special hematological investigations were not being offered and Giemsa stained peripheral blood or bone marrow aspirations smears were the only diagnostic means in the beginning. After recruiting a laboratory scientist, a small CCC hematology laboratory was opened, starting with a microscope and manual Giemsa, Sudan Black and Myeloperoxidase staining only. The necessity of blood tests prior to every cycle of chemotherapy, the investment in blood count and biochemistry machines was reasonable and empowered the small laboratory to work economically and sustainably through reimbursements of standard biochemistry and blood count tests.

A Flow Fluorescence-activated Cell Sorting (FACS) machine (“FACScalibur”, BD Medical) was installed in the main hospital laboratory for CD4 counts of HIV patients. These machines are widely distributed in Sub Saharan Africa by the PEPFAR (The United States President´s Emergency Plan for AIDS relief) program for CD4 counts. Due to the favorable use of viral load instead of CD4 counts in more recent times, the machine was mostly not utilized, which gave CCC the chance to procure reagents and reactivate the FACS for leukemia and lymphoma diagnostics, without investing in a new machine.

The same approach was used with the polymerase-chain-reactions (PCR). The widely available GeneXpert (Cepheid) machines in Sub-Saharan Africa for tuberculosis testing, can now be adapted for BCR-ABL fusion gene diagnostics. Just as with the FACS machine, procurement of cartridges remained the only costs for the Centre.

Collaboration and partnering with various international organizations, was—and still is—a mainstay of the CCC´s functionality. The MAX Foundation (former Gleevec International Patient Assistance Program) partnered with CCC in August 2018 and continues to deliver first and second-line therapies for CML patients on a no cost basis and supports procurement of discounted PCR cartridges as well. The German Institute for Medical Mission (DIFÄM), a long-standing partner of KCMC, expanded its support to the CCC with the procurement of standard chemotherapies and facilitates the Centre with protective garments for safe mixing and administering of cytotoxic medications. In addition, annual hands-on trainings on site, is conducted by an experienced pharmacist from DIFÄM. FCCT and the German-Bavarian Lutheran Church are strong partners for donations of equipment and funding of various programs of the CCC (palliative care delivery, prevention and awareness programs). Through the American Society of Clinical Pathology (ASCP), the Pathology Department of KCMC was equipped with a telepathology machine and immunohistochemistry reagents.

The Centre has received several volunteers throughout the years. Especially senior experts with decades of experience in their respective fields are of great value for the few specialists in the Centre to discuss treatment protocols and approaches adapted to the Tanzanian setting. On the other hand, the Centre sends staff abroad, for exposure to high resource settings where ideas can be gained and implemented in an adapted way for the CCC setting.

**Fig 4:**
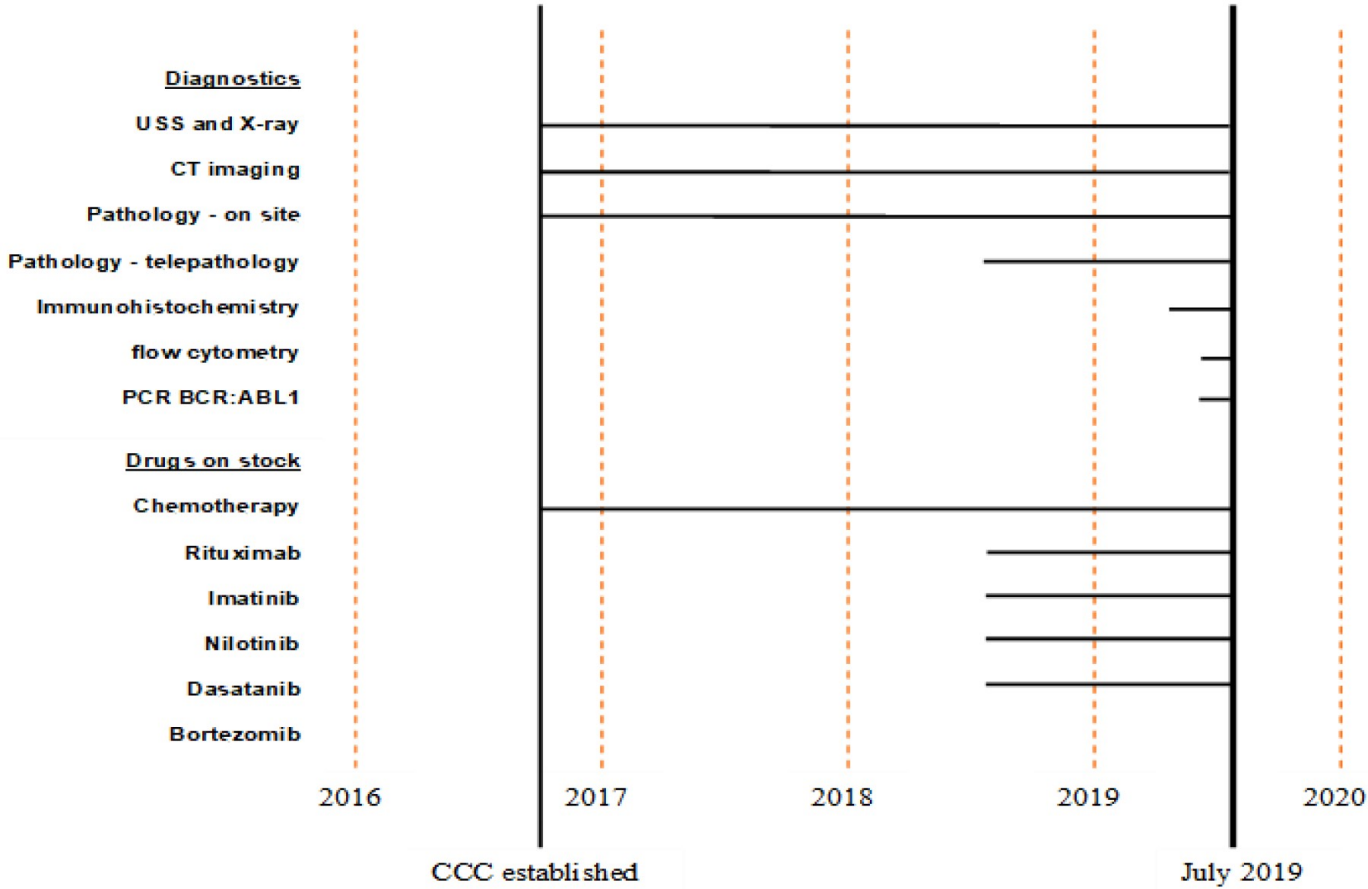
Diagnostics and medication on stock at the CCC since its establishment.

Figs 3 and 4 demonstrate how staff recruitment, diagnostic means and treatment options have evolved at CCC since its establishment in 2016.

## Discussion

Prior to 2016, hematological malignancies were underrepresented in the Kilimanjaro Cancer registry. Lack of formal diagnosis in the absence of available services, and limitations in case reporting were likely contributing factors. Our data suggests that hematological malignancies are a significant problem within this East African population and are typically diagnosed at a younger age and at a later stage than in patients in high income settings. Additionally, Tanzania has one of the fastest growing populations and with this incidence will undoubtedly increase. Nonetheless, it is possible to establish a well-functioning hematology service within a low resource environment by using cooperation and already existing infrastructure.

### Age at presentation

Average age at presentation in patients with MM, HL, CML and CLL were seen to be at least ten years lower than ages quoted by American and European literature. The median age for MM diagnosis in high resource settings is usually around 70 years of age with around 2% of cases occurring in those less than 40 years. In our cohort, median age at presentation was 58 years [7]. Similar figures have been documented in retrospective studies conducted in both Nigeria and Cameroon where median ages of 62 and 57 years have been quoted [8,9]. Interestingly however, in American cohorts although disease seems to be more aggressive in black patients, with shorter overall survival, no racial disparity was noted in age of onset [10,11].

A median age of 56 was recorded in patients with CLL in this cohort, which again compares to higher ages of 65–70 years in European and Northern American publications [12,13,14]. This younger age appears to correlate with an emerging pattern in African patients with average ages at onset of 56, 55 and 61 in Nigerian, Ethiopian and Senegalese cohorts respectively [15,16,17]. Unlike in MM however, younger age at presentation has also been reported in black patients in high income settings [18].

In CML, median age of 42 years was noted comparing to 66 in European and North American publications [12,13]. Again, similar trends are emerging, median age of 42 years at diagnosis was reported in a recent Senegalese cohort [19] and a mean difference of -7 years was seen between white and black patients in an American study [20].

Finally, in the case of Hodgkin lymphoma, same trends of earlier presentation have been seen in black patients and additionally a less apparent bimodal age distribution has been reported. Only the early peak in the 15–24 years old patients was observed in our cohort fitting with similar epidemiological data [21].

Although we can see that earlier age of onset does appear to be seen in black patients in high income settings in the case of CLL, CML and HL, these ages are still significantly higher than those reported in literature from Sub Saharan African cohorts [12,18,21]. Undoubtedly therefore genetics are likely to play a role, however environmental factors must be at play given the geographical disparity.

There are a number of possible explanations for these findings that have been proposed. Infection is likely to play a role with higher rates of HIV and EBV exposure affecting rates of lymphoma in younger patients [22]. There has also been a demonstrated link between multiple myeloma and EBV however impact on age of onset has not been determined [23]. In the case of our CLL patient population, the use of pesticides and artificial fertilizer might contribute to the number of CLL/SLL [24,25], taking into consideration that Kilimanjaro Region is known for a large use of pesticides [26], partly exceeding the WHO permissible limits, at least in the past [27]. However further research is required to evaluate the association between these factors and to identify other potential etiological causes.

### Late stage presentation

Of interest is that these differences in age at presentation between high and low income settings are likely an undervaluation given the additional late stage at diagnosis also observed in this cohort. Late presentation has been well documented in the case of malignancies in low income settings [20]. Multiple factors have been suggested as contributing factors, including financial and geographical constraints. For patients living up to hundreds of kilometers from the center, or those experiencing financial burden, access to healthcare is limited [28]. In addition, lack of education about cancer amongst patients and health care workers as well [29,30] results in patients seeking health care elsewhere such as traditional healers, or the patients are misdiagnosed and treated incorrectly. For example, we experienced a lot of lymphoma cases that have been initially treated unsuccessfully for tuberculosis lymphadenitis for 6 months, before referral for biopsy consideration was made.

The median age at presentation is therefore estimated to be even younger if patients would present at a similar stage to those in high income settings. This is reflected in our cohort with 91% of HL cases and 81% of NHL cases presenting with Ann-Arbor stage 3 or 4 disease and 80% of all documented CLL cases presented in Binet stage C. Almost all patients with MM (93%) had bone lesions at time of diagnosis and hence presented in Salmon and Durie stage 3.

### Diagnostics

Diagnosis and classification of hematological malignancy is becoming increasingly sophisticated as technology advances. This allows the development of new risk classifications and personalized medicine. But the costs of these diagnostics make their application in resource limited settings restricted.

Diagnosis at CCC is based on simple and cheap techniques ranging from morphology of the blood smear, bone marrow aspiration cytology, trephine histopathology and lymph node biopsies. The more expensive diagnostic means PCR and FACS was introduced by using existing infrastructure. This approach does not only lower capital costs, it avoids underutilization of the machines that lead to higher cost-per-test [31]. Furthermore, in the case of BCR-ABL PCR, it has reduced the costs for patients to 50% compared with the costs at private run laboratories and shortened the time to definitive diagnosis tremendously. Mendizabal et al. and Faye et al. reported about delays from diagnosis to treatment initiation, which can last more than 1 year due to financial constrains in paying for necessary diagnostic tests [32,33]. At CCC, the average time for definitive CML diagnosis is now less than 1 week.

Cooperating with international professional societies play a role in maintaining high quality results and for example telepathology gives (hemato-) pathologists an instant feedback on their findings and can contribute to quality assurance [34].

Apart from internal issues at the respective hospitals themselves, laboratory diagnostics in low resource settings face challenges like lack of reliable local or national vendors for quality reagents and consumables and slow and costly customs clearance process for international procurement [35,36] which leads to recurrent out of stock situations.

### Treatment

Tanzania’s National Health Insurance Fund (NHIF) covers most of the patient’s chemotherapy treatment, including three antibodies (Rituximab, Trastuzumab and Bevacizumab), but the overall coverage is low, reaching only 7% of Tanzania´s population [37]. While the Community Health Fund covers 25% of the population, chemotherapies are not covered. Even though, through the governmental Medical Store Department, cancer medication can be obtained free of costs, procurement from this source of medication remains widely incomplete, hence CCC faces many out of stock situations. Therefore, deliveries through the private sector and donations through direct supply from DIFÄM, MAX Foundation and—for pediatric patients—from the Pediatric Department of the Muhimbili National Hospital (MNH), remain main sources.

The use of generic medicines and biosimilars should always be considered for cost savings and guidelines should be followed using the cheapest available medication, if applicable (e.g. starting Imatinib instead Nilotinib as first line treatment in CML patients).

For uninsured patients, the CCC social worker assesses the financial situation of each individual and obtains social support for those patients who cannot cover the cost of therapy, although this naturally cannot cover the more expensive treatments.

To date, CCC provides mostly the standard chemotherapy treatment regimens for hematological malignancies as first line treatments. Addition of Rituximab depends on drug availability, patient insurance status and financial ability of the patient. Second line treatments are also offered following international guidelines.

Despite increasing access to chemotherapies and monoclonal antibodies, challenges still persist. Transportation is a major limitation for many with some patients living up to two days drive from CCC. Hostel accommodation can be provided free of charge, however this requires complete relocation resulting in economic limitations for the patient and the greater family unit [38]. Even with patients living closer to the centre, limitations with infrastructure and transport particularly during rainy season can pose issues and often leads to delays or loss to follow up [38]. Similarly, for some patients, seeking urgent medical attention for example in the event of neutropenic fever is difficult, although this is hard to fully assess.

Management of the acute leukemias pose their own unique challenges. Delivering treatment often becomes unrealistic when considering barriers including blood product availability [39], patient isolation, broad spectrum antibiotic availability and the financial burden to uninsured patients. However, as the CCC expands and with plans in place for a dedicated oncology inpatient unit, many of these challenges will be mitigated. Induction therapies have been offered to AML patients, but infections during nadir pose an unsolved threat to date. Pediatric acute leukemias are referred to MNH as CCC is lacking a pediatric haemato-oncologist currently.

### Limitations

It is likely a number of cases have not been registered in the cancer registry. Many of the acute leukemias for example are not treated due to factors described and often a formal diagnosis will not be made if death occurs early in admission due to the limited post-mortem service. Secondly, numbers for pediatric cases in particular are likely underrepresented due to direct referral to the Pediatric Department of MNH without passing through the CCC.

With regards to staging, what has been recognized is that in the early stage of the cancer registry recording of disease stage was sporadic and it has been difficult to chase paper files as the CCC moved to an electronic system. Ongoing advancements in the registration of cancer cases will lead to more accurate data in the future.

## Conclusions

Hematological malignancies in Northern Tanzania occur generally at a younger age and a higher stage than in Europe or North America and it can be assumed that in particular infections play a key role herein.

Establishing services for the diagnosis and treatment of hematological malignancies in low income settings is not without its many challenges. However, the example of the establishment of the CCC at KCMC displays a way forward to close this service gap by utilizing and adapting existing infrastructures and machines, partnering with national and international organizations and universities. Data collection and accurate analysis will aid better understanding of hematological malignancies in the Tanzanian setting and allows better projecting and planning for future strategies to respond to the growing burden of hematological cancer diseases.
